# Personalized medicine – where do we stand? Pouring some water into wine: a realistic perspective

**DOI:** 10.3325/cmj.2012.53.314

**Published:** 2012-08

**Authors:** Ursula Gundert-Remy, Aleksandar Dimovski, Srećko Gajović

**Affiliations:** 1 Charité Universitätsmedizin Berlin, Institute for Clinical Pharmacology and Toxicology, Berlin, Germany *ursula.gundert-remy@charite.de*; 2 Department of Biomolecular Sciences, Institute of Pharmaceutical Chemistry, Faculty of Pharmacy, University St. Cyril and Methodius, Skopje, Macedonia, F.Y.R.; 3 University of Zagreb School of Medicine, Croatian Institute for Brain Research, Zagreb, Croatia

## Abstract

**Abstract:**

Reviewing the past and the present status of personalized medicine, the hope and promise from several years ago was critically compared to what is really achieved to tailor the drug treatment according to the patient’s individuality. The basis for consideration is what we know about the variant of the disease the patient is suffering from, and about the mechanisms influencing the plasma concentration-time profile, such as activity of metabolizing enzymes and transporters. In cancer treatment, drugs are currently selected regarding molecular properties of the cancer tissue, eg, expressing receptors such as HER2 receptor. Currently diagnostic tests are available allowing to detect somatic cell mutations that can be used to guide drug selection. Unfortunately, tumor heterogeneity and developing resistance by further mutations may limit the success of the therapy determined by molecular diagnostics. The present status can be described that in drug kinetics we know the influencing factors and we understand the mechanisms. However, only in a few cases the genetic background is the main determinant of kinetic variability, and environmental and other factors have an additional important role. Therefore, much more has to be done before we can translate the accumulating knowledge into a benefit for the patient. Only then, we can speak about personalized medicine.

## What is personalized medicine?

Personalized medicine is an expression embracing ideas and a concept of making the treatment of a patient as individualized as possible. The therapy should be guided by clinical, genetic, genomic, and environmental information, which is different for every patient. The data offered are about the underlying mechanisms of the patient’s disease, and can be used for drug selection (“selecting the right drug”) or to determine the appropriate dose (“selecting the right dose”). The individual response to drugs depends not only on the mechanisms of the disease (pharmacodynamics), but in addition on the handling of the drug by the patient (pharmacokinetics).

In 2002, the successful sequencing of the human genome was greeted with much enthusiasm. It was announced that the era of molecular medicine has begun and scientists and some physicians were promising that the field of personalized medicine would come at a fast pace. The hopes of many patients, which the high flying promises have provoked have still to be fulfilled. In addition, it is to be noted that this progress will not come without costs in the health care system and that there are financial interests intertwined with the science and with the good intentions to provide the patients with the best medical care they deserve.

Personalized medicine also encompasses the management of patients’ personal data and medical information by information and communication technology (ICT) (1,2). It should however be noticed that frequently the medical doctors and patients are reluctant to make this information available on ITC platforms because of fear that the data could leak into the public domain. In some countries, there may be unwanted corollaries if the information is available with respect to employment and health insurance. Hence, the progress might come with some expenses that have to be weighed against the benefits it brings with it.

## How it started –pharmacokinetic observations were first

In the 1950s, it was clinically observed that patients differed concerning their reactions toward drugs, including the side effects. The first description of an inherited defect concerned the muscle relaxant succinylcholine. The duration of this drug’s action is determined by hydrolysis. Subjects homozygous for a gene encoding an atypical form of the enzyme mediating the hydrolysis have a prolonged drug-induced muscle paralysis. At this time, clinical observation and family tree investigation built the tool to identify inheritance. The tuberculostatic isoniazid is metabolized by N-acetylation. It became obvious that side effects were related to prolonged half-life and it was identified that the defect in N-acetylation activity was hereditary. The same pattern could be described for the antihypertensive drug hydralazine and the antiarrythmic drug procainamide (3,4). In the mid 1970s, findings of a new drug metabolizing enzyme defect drew the attention toward hydroxylation of drugs (5-7). Later, it was found out that hydroxylation of drugs was mediated by the cytochrome P450 enzyme (CYP) system, the most important system in terms of drug metabolism in humans. The molecular basis for the observed defects was only elucidated at the end of the 1980s by Gonzales et al (8) and Kimura et al (9), showing that the phenotype of poor metabolizer was characterized by mutant alleles leading to lacking or inactive enzyme. The mutations differed in patients with the same phenotype. The clinical importance of the findings and their implications for drug development was discussed at this time. It was obvious that clinically relevant changes of drug response and possible side effects were expected only with drugs having a steep concentration-response relationship and/or a narrow therapeutic window (10). The prevalence of the slow metabolizer phenotype of the debrisoquine type was determined to be 7.40% in the European population based on 8764 determinations, consistent with a gene frequency of 0.27. The debrisoquine phenotype was later attributed to the gene regulating the expression of cytochrome (CYP) 2 D6. The overall mean of the phenotype of slow metabolizers of mephenytoin was 3.52%, corresponding to a gene frequency of 0.19 (11). The phenotype was later attributed to CYP 2C19. These data show that the polymorphism of hydroxylation is based on the prevalence and the gene frequency in the population. It later became obvious that not only gene defects with an impaired function are present in the population but also gene duplications with the consequence of an enhanced metabolic capacity. The consequences for dosing are obvious: in patients with inactive enzyme (“poor metabolizers”) the dose of the drug must be reduced and in patients with gene amplification and enhanced metabolic capacity (“ultrarapid metabolizers”) the dose must be increased (12).

## What is the present status in pharmacokinetics?

Presently, in the therapeutic reality there are only few examples that demonstrate a clear correlation between therapeutic outcome and metabolic capacity. In a study of psychiatric patients treated with the antidepressant venlafaxine ultra rapid, extensive, intermediate, and poor metabolizers of CYP2D6 were identified. Poor metabolizers had more side effects in comparison with other patients (*P* < 0.005), still the therapeutic efficacy did not significantly differ between the different phenotypes (13). The poor predictivity of therapeutic outcome guided by only one factor, namely activity of the metabolizing enzyme, points to the fact that the variability in metabolism and drug response is due to multiple factors that contribute to the observed variability. One single factor explains only a certain part of the total variability. Hence, the approach of identifying several factors/markers is better suited to explain variability. One recent example is given by Sistonen et al (14), who identified genetic markers predictive of central nervous system depression in 111 breastfeeding mothers using codeine and their infants. A genetic model combining the maternal genotypes of CYP2D6 and ABCB1 transporter predicted 87% of the infant and maternal central nervous system depression cases with a sensitivity of 80% and a specificity of 87%. In addition, the anticoagulant drug warfarin is a further example. It is widely prescribed for therapeutic anticoagulation. Patients vary widely (20-fold) in the dose needed to achieve an appropriate level of coagulation parameters to prevent either too low (risking under-treatment) or too high doses (risking severe bleeding). In a genome-wide association study, Takeuchi et al (15) confirmed that two genes (*VKORC1*, *CYP2C9*) explained about 40% of the variability in warfarin dose. They discovered a new gene (*CYP4F2*) contributing to 1%-2% of the variability. Unfortunately, clinical trials assessing patient benefit from individualized dose forecasting based on the patient's genetic makeup at *VKORC1*, *CYP2C9,* and *CYP4F2* have not yet provided sufficient evidence to support the use of pharmacogenetics to guide warfarin therapy (16). Only the time to achieve appropriate anticoagulation was shorter when dosing was guided by CYP 2C9 genotyping (17). Further clinical trials are needed to define how warfarin pharmacogenetics could contribute to a better dosing in clinical practice. The rather confusing situation with clopidogrel, an antiplatelet acting substance, has been elucidated by Johnson et al (18). Clopidogrel is a prodrug that needs activation by CYP2C19 dependent metabolism. Carriers of the CYP2C19 *2 allele (heterozygotes and homozygotes) have lower active metabolite plasma concentrations and less antiplatelet effect. Johnson et al (18) analyzed available studies and came to the conclusion based on their analysis of the patients for whom clopidogrel provides the greatest benefit: namely, patients after percutaneous coronary intervention, which includes stenting, do also profit from CYP2C19 genotyping prior to dosing. In patients treated for other indications, the clinical benefit was independent from the metabolizer status. Harmze et al (19) modified this view looking at side effects. They found that patients with the CYP2C19*1/*17 and *17/*17 diplotype had an increased risk of major bleeding events after coronary stenting than patients with the *1/*1, the “wild” genotype. Testing before treatment with clopidogrel to identify the appropriate individual dose is recommended by American Food and Drug Administration (FDA) but not by the European Medical Agency (EMA) (20).

## Side effects of drugs and genetic markers

It has been known for a long time that the genetic disposition is important for the development of side effects of drugs (21,22). Liver injury is one of the most important side effects because it is severe and even life threatening. In genome-wide association studies, the human leukocyte antigen (HLA) system has been identified to play a role in eliciting major and clinically important side effects. Until now, the knowledge on the genetic basis of the drugs’ side effect has only partially transformed into the clinical handling of these drugs. For example, testing is required before abacavir is given to a patient (20). However, testing before prescribing was not required for other drugs until now, although the information is mentioned in the drugs’ information sheet (so-called labeling) at least for drugs approved by FDA.

## What is the status in pharmacodynamics?

The effects of drugs vary widely among a population. This is not only because of variability in the kinetics of the drug but also because of the underlying mechanism responsible for the disease that may be different irrespective of the identical phenotype. It should be noted that disease may be due to mutations in the germ cell line and potential factors for hypertension may be one of the examples. In cancer, however, the mutations may concern not the germ cell but the somatic cell line, for example in colo-rectal carcinoma (CRC). We address here two major therapeutic fields, cardiovascular diseases and cancer, to explore the contribution of genome analysis in the selection of the appropriate drug for the individual patient.

### Cardiovascular diseases

Genomics in the cardiovascular field is directed toward understanding biological mechanisms of diseases and translating that knowledge to select the appropriate drug for the individual. During the past 5 years, hundreds of cardiovascular loci have been discovered. Genome-wide association study has been undertaken by the international consortium for blood pressure and it identified in 200 000 Europeans 29 single-nucleotide polymorphisms (SNPs) at 28 loci associated with regulation of the blood pressure (23). It is understandable that this information is of interest for the possible development of new drugs targeting specific regulation mechanisms. Molecular findings, however, have not yet found the way into clinical practice. The Seventh Report of the Joint National Committee on Prevention (*www.nhlbi.nih.gov/guidelines/hypertension/)* discusses clinically determined criteria helpful for selecting the appropriate antihypertensive drug, such as concomitant renal insufficiency, diabetes, and left ventricular hypertrophy. Currently only phenotypic factors but no genotypic factors influence the selection of antihypertensive drugs for the individual patient.

Pharmacogenomics might explain the variation in drug efficacy and more consideration needs to be given to the clinical context to define where pharmacogenomics would be an additional tool to monitor or predict therapeutic success (24). As, at present, the place of pharmacogenomics in cardiovascular medicine is not defined, further studies are needed recruiting tens of thousands of patients with cardiovascular disease that combine tests of genome-wide association with sequencing. The genomic studies have to be supplemented by functional studies aimed to characterize molecular and cellular pathways. It should also be noted that there is more than the genome that influences treatment outcome, namely clinical, biological, or environmental factors that have to be meaningfully integrated to support personalized decision making in cardiology (25).

### Cancer

The field of oncology is the area in medicine where genomic data and information is used on a daily basis. Here we can present several well established examples of the advantage of its use in identifying somatic mutations in the genotype of the tumor that are strong determinants of drug response.

*Breast cancer.* The HER2 (also called ErbB-2) receptor belongs to the epidermal growth factor receptor family of receptors characterized by an extracellular ligand binding domain, a transmembrane domain, and an intracellular domain. Epidermal growth factor receptors have an activity as plasma membrane-bound receptor tyrosine kinases. Their intracellular domain can interact with a multitude of signaling molecules in the cell. Signaling through HER2 or other members of the receptor family promotes cell proliferation and opposes cell death by apoptosis. In normal life, signaling through this pathway is tightly regulated to prevent uncontrolled cell growth. Amplification of the gene regulating the receptor protein or gene overexpression is seen in 15%-20% of breast cancers. Gene amplification or overexpression is a prognostic marker and indicates a poor outcome of the disease (26). During the past decade, treatment, specifically targeted at HER2, has improved disease-free survival in patients with breast cancers that overexpress HER2 but it has not convincingly increased the overall survival (27). Nevertheless testing is necessary, before treatment is initiated, in order to predict whether drugs directed toward HER2 will have a chance to be effective or whether the treatment will only cause side effects.

Very recently it has been demonstrated that acting on the same target by adding pertuzumab to a treatment with trastuzumab and docetaxel does improve progression-free survival in patients with HER2 positive metastatic breast cancer (control median: 12.4 months; addition of pertuzumab 18.5 months). However, the positive influence on overall survival of the patients is still to be documented (28).

*Colo-rectal cancer.* Another example where diagnostic testing is used is the anticancer therapy directed toward epidermal growth factor receptor (EGFR). Similar to HER2, the EGFR receptor is on the surface of cancer cells. Monoclonal antibodies have been approved to treat EGFR-expressing late-stage colorectal cancer (CRC) in patients who had become resistant to chemotherapy. It has been found out that the drug is not effective in patients whose tumors have a mutated KRAS gene, which is associated with resistance to drugs that are anti-epidermal growth factor receptor (EGFR) antibodies. The tumors of patients with metastatic colorectal cancer are now profiled for seven KRAS mutations before receiving monoclonal antibodies. Polymerase chain reaction-based kits (eg, Therascreen, Quiagen) can provide information about the KRAS gene mutation in patients whose CRC has metastasized. These tests have been approved recently by FDA to determine if the absence of a gene mutation would indicate the treatment with a monoclonal antibody. In one clinical study with cetuximab, in patients whose tumors did not have a KRAS mutation, median survival was 8.6 months compared with 5 months in the control group. In patients with a KRAS mutation, median survival was similar in those who received the drug as in the control group (4.8 months and 4.6 months, respectively). Hence, testing is helpful for predicting the outcome. However, we have to admit that we still wait for therapeutic breakthroughs as even in the most favorable case survival was prolonged for a few months only. In a recent study, other mutations, namely BRAF, NRAS, and PIK3CA exon 20 mutations, were also found to be associated with a low response rate. From this study, it can be concluded that additional genotyping of BRAF, NRAS, and PIK3CA exon 20 mutations in a KRAS wild-type population may help to identify patients with a good therapeutic outcome (29).

## Approval of tests together with the drug

Two further tests to increase the prediction of tumor response were approved. Anaplastic lymphoma kinase (ALK) is the genetic basis for a receptor tyrosine kinase involved in cell growth regulation. Rearrangement in this gene (ALK+) has a role in the oncogenesis of non-small cell lung carcinomas (NSCLs), especially adenocarcinomas. The ALK inhibitor crizotinib was approved in August 2011 by the FDA for treating late-stage NSCLCs that are ALK+ together with the diagnostic test to enable appropriate “individualized” therapy. However, meanwhile acquired crizotinib resistance in ALK+ non-small cell lung carcinomas was observed (30).

Another key finding, which explains the limited success of individualized therapy besides acquired resistance, is that different regions of the tumor have different mutations. Single tumor-biopsy samples can therefore lead to underestimate intra-tumor heterogeneity. Tumor heterogeneity may enhance adaptation of the tumor through Darwinian selection and lead to therapeutic failure. In addition, alterations of the genes by epigenetic mechanisms and further changes in signal transduction may be important for the survival of the tumor. Based on these findings and results we have to admit that the concept of directing therapy based on genetic tumor markers is probably too simple.

The lesson to be learned is that genetic changes have to be expected within some months of therapy. If we can identify the genes that are affected by Darwinian selection we could be able to make them targets for drug development (31,32).

## What is the perspective?

### On the scientific level

In 2002, the human genome project was completed. Since that time, the techniques used in genomics have tremendously progressed. Methodological advances in statistics have yielded hundreds of confirmed associations between genes and disease. We expect that improvements in “next generation” sequencing will soon be available, making it feasible that people will be able to carry their genome on a memory stick. However, at present it is yet unclear whether anyone would want to do so.

Obviously, the present approaches are conceptually too simple to cover the biological reality. The human nuclear genome is only apparently a simple construct since it contains 3.2 billion nucleotides. A complex system guides access to the double-stranded DNA by regulating synthesis and gene expression in response to internal and external stimuli. Variability and susceptibility is not only determined by DNA sequence variants (DSVs) but by mechanisms that govern the expression of a phenotype, such as histone modifications, microRNAs, long noncoding RNAs, epigenetics, splice variants, and posttranslational modifications of the encoded proteins. At present, we only partially understand how the environmental factors come into the play as the mechanisms of epigenetic modifications were unfolded (32,33). Furthermore, gene-gene interactions were overlooked in the past because of the computational burden of interactions assessment, and it is still not in the focus of genome-wide association studies (34).

Thus, a complex phenotype of an individual is the consequence of complex interactions of a large number of genetic and non-genetic determining factors, which we currently are not able to decipher.

The scientific committee of the National Cancer Institute in the US has recently given recommendations to accelerate translation from basic science into the clinical application. The recommendations are directed toward gaining more knowledge on the relationship between germ line genetic make-up, somatic genetic changes, phenotype, and features related to cancer treatment response and adverse events (35).

### On the level of dissemination

In order that personalized medicine can be used effectively by health care providers, and the patients, the findings in the field must be translated into information when the diagnostic tests and targeted therapies are right in place. This information is given occasionally, eg, in testing patients genetically to determine their likelihood of having a serious adverse reaction to various cancer drugs. The information on genetic markers for drug and dose selection in the drug data sheet is provided by FDA.

However, the results of a recent study in the US (36) indicate that even physicians feel not well informed about the clinical value, availability, and interpretation of pharmacogenomic tests. A recent survey of surgeons in the United Kingdom found, worryingly, that only half of patients with invasive cancer had a HER2 result available when treatment was initially discussed (37). The findings highlight the need for a more effective physician education and better information of the administration responsible for managing to timely provide the testing results.

Unfortunately, most information dissemination found on the internet expresses high flying promises. It would be better to provide information on what we know and what we do not know yet and what benefit a patient really can expect for what cost.

## Conclusion

The molecular basis of variability in effective drug doses was understood several decades ago. Understanding, however, does not transfer automatically in therapeutically important improvements. Whereas the concept of testing for molecular changes to explain metabolic differences is accepted, we have to note that the clinical influence of a changed metabolism is modest in most of the cases. Somatic mutations are important for detecting whether the patient will respond to new drugs targeted for tumor specific molecular features. Success is limited by tumor heterogeneity, which is not easily detected and by development of tumor cell resistance at the molecular and cellular level. There is a long way to go to fight these obstacles. The concept of personalized medicine is intellectually attractive and scientists are convinced that this is the way forward. However, dissemination of the current status instead of unrealistic promises will be a cornerstone for the acceptance by doctors and patients alike.
